# Seasonal Variations of the Activity of Antioxidant Defense Enzymes in the Red Mullet (*Mullus barbatus* l.) from the Adriatic Sea

**DOI:** 10.3390/md8030413

**Published:** 2010-02-26

**Authors:** Sladjan Z. Pavlović, Slavica S. Borković Mitić, Tijana B. Radovanović, Branka R. Perendija, Svetlana G. Despotović, Jelena P. Gavrić, Zorica S. Saičić

**Affiliations:** Department of Physiology, Institute for Biological Research “Siniša Stanković”, University of Belgrade, 11060 Belgrade, Serbia; E-Mails: sladjan@ibiss.bg.ac.rs (S.Z.P.); borkos@ibiss.bg.ac.rs (S.S.B.M.); tijana@ibiss.bg.ac.rs (T.B.R.); perendija@ibiss.bg.ac.rs (B.R.P.); despot@ibiss.bg.ac.rs (S.G.D.); jelena.gavric@ibiss.bg.ac.rs (J.P.G.)

**Keywords:** red mullet, oxidative stress, liver, white muscle, Adriatic Sea

## Abstract

This study investigated seasonal variations of antioxidant defense enzyme activities: total, manganese, copper zinc containing superoxide dismutase (Tot SOD, Mn SOD, CuZn SOD), catalase (CAT), glutathione peroxidase (GSH-Px), glutathione reductase (GR) and biotransformation phase II enzyme glutathione-S-transferase (GST) activity in the liver and white muscle of red mullet (*Mullus barbatus*). The investigations were performed in winter and spring at two localities: Near Bar (NB) and Estuary of the River Bojana (EB) in the Southern Adriatic Sea. At both sites, Mn SOD, GSH-Px, GR and GST activities decreased in the liver in spring. In the white muscle, activities of Mn SOD, GSH-Px, GR and GST in NB decreased in spring. GR decreased in spring in EB, while CAT activity was higher in spring at both sites. The results of Principal Component Analysis (PCA) based on correlations indicated a clear separation of various sampling periods for both investigated tissues and a marked difference between two seasons. Our study is the first report on antioxidant defense enzyme activities in the red mullet in the Southern Adriatic Sea. It indicates that seasonal variations of antioxidant defense enzyme activities should be used in further biomonitoring studies in fish species.

## 1. Introduction

Fish as species are on top of the aquatic food chain; as vertebrates, they strongly respond to stress conditions [1]. Therefore, they are often used as indicator species of pollutant exposure in the aquatic environment. Evaluation of seasonal variations in biomarkers and determination of basal levels in model organisms constitute a research strategy that is widely recommended today. This effort overcomes the difficulties involved in field studies, integrating variations in many natural stressors and evaluating the effects of chemical pollution [2].

The use of fish in environmental monitoring has become increasingly important in recent years in the investigation of natural variability, as well as anthropogenic substances, many of which function as prooxidants, accumulating in aquatic environments [3]. Many studies of antioxidant defense enzyme activities in aquatic organisms, particularly in fish, were designed to provide data for comparative studies or to examine the effects of environmental influences, e.g. diet, seasonal variation and the influence of contaminants [4,5]. Many environmental factors induce the production of reactive oxygen species (ROS). As temperature-dependent organisms, most fishes must routinely cope with fluctuations in environmental temperature and in the metabolic rate and consequently with oscillations in ROS levels [6]. Therefore, ROS generation, oxidation rates and antioxidant status are directly related to ambient temperature or metabolic activity [7]. Studies on oxidative stress biomarkers related to seasonal changes in poikilothermic organisms revealed their strong relationship with metabolic demands [7,8]. It means that lower metabolic rate is accompanied with lower antioxidative defense, but the role of individual components in achievement of homeostasis seams to be different and integrated in antioxidative defense system. Fish are exposed to daily and/or seasonal changes in both water temperatures and oxygen availability; variations in the activity of oxidative stress biomarkers have been demonstrated in several studies and proposed as biomarkers of pollutant-mediated oxidative stress [9,10].

Several classes of pollutants, including trace metals and organic compounds, are known to enhance the formation of ROS resulting from xenobiotic redox cycling [3]. A battery of enzymes and molecules plays important roles in detoxifying xenobiotics and ROS, thus it has been applied as a biomarker for environmental risks in fish [8,11]. The use of a battery of biomarkers will provide a more complete picture of various effects on oxidative stress in the cells of an organism [3].

Phase I and phase II biotransformation reactions are of great importance in the understanding of metabolism of endogenous molecules and transformation of xenobiotics and drugs in fish and other species [12]. The main enzymes that detoxify ROS in all organisms are functionally divided into antioxidant defense enzyme activities (superoxide dismutase-SOD, catalase-CAT, glutathione peroxidase-GSH-Px and glutathione reductase-GR) and biotransformation phase II components (for instance, glutathione-S-transferase-GST and reduced/oxidized glutathione) [8]. Our previous report considered glutathione-dependent and other antioxidant defense enzymes as markers for oxidative stress in fish [9,10]. Depending on the availability of nutrients, reproductive status, season-related growth rate and other factors, the activity of antioxidant defense enzymes and other biomarkers fluctuates significantly throughout the year [13]. Some aspects of seasonal variations in antioxidant defense were observed in tissues of thin-lip gray mullet (*Liza ramada*) [9], mussel (*Mytilus galloprovincialis*) [5,14], horse mussels (*Modiolus modiolus*) [15], blue mussels (*Mytilus edulis*) [16] and in the digestive gland of brown mussels (*Perna perna*) [17].

The benthic fish, red mullet (*Mullus barbatus* L.) was chosen as the bioindicator species because it is a territorial fish of commercial interest in fisheries and aquaculture, which has been used in several studies of coastal pollution monitoring. Red mullet is a perciform species that feeds mainly on zooplankton, benthic organisms and detritus. Due to its close association with sediments and wide geographical distribution, the red mullet can be considered a key indicator species for the Adriatic Sea [18,19].

The aim of this study was to explore seasonal variations in the activity of the antioxidant defense enzymes: total superoxide dismutase (Tot SOD), manganese containing superoxide dismutase (Mn SOD), copper zinc containing superoxide dismutase (CuZn SOD), (EC 1.15.1.1), catalase (CAT, EC 1.11.1.6), glutathione peroxidase (GSH-Px, EC 1.11.1.9), glutathione reductase (GR, EC 1.6.4.2), and the activity of biotransformation phase II enzyme glutathione-S-transferase (GST, EC 2.5.1.18) in the liver and white muscle of red mullet (*Mullus barbatus*) in winter and spring at the localities: Near Bar (NB) and Estuary of the River Bojana (EB) in the Southern Adriatic Sea.

## 2. Results and Discussion

The studied areas (Near Bar and Estuary of the River Bojana in the Southern Adriatic Sea) were selected because both receive extensive industrial and urban wastewater discharges. They have similar climates, with the lowest mean water temperature of 12.5 °C in February and the highest of 20.4 °C in August. Estuary of the River Bojana is characterized by a higher inflow of freshwater than Near Bar locality. The mean sea depth at Near Bar is 70 m and at Estuary of the River Bojana is 30 m. The bottoms of the biotopes are covered with thick stratum of fine terrigenous mud with particles of detritus. The sea currents at both localities are very irregular; in the summer they are slight, while in winter they are very strong [20].

**Figure 1:**
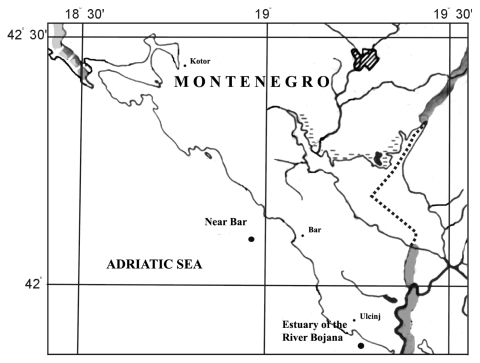
The geographical position of the localities of Near Bar (NB) and Estuary of the River Bojana (EB) in the Southern Adriatic Sea.

The geographical position of the investigated localities is shown in Figure 1.

The data concerning physic-chemical characteristics of seawater are presented in Table 1. The results obtained show that water temperature was significantly higher in spring in comparison to winter at both investigated localities. Other environmental parameters (salinity, oxygen concentration and oxygen saturation) were similar between the two localities in each season.

Total protein concentration in the liver and white muscle of *M. barbatus* at both sites in winter and spring is shown in Table 2. The presented results show that total protein concentration was significantly higher in the liver than in white muscle at both sites and seasons. Total protein concentration was significantly lower in the fish liver from Estuary of the River Bojana in spring in respect to winter (p < 0.05). In contrast, total protein concentration was markedly higher in the white muscle from near Bar in spring in comparison to winter (p < 0.05). These data suggest different metabolic activity of these two tissues in respect to season and probably depend on food availability and feeding behavior.

**Figure 2:**
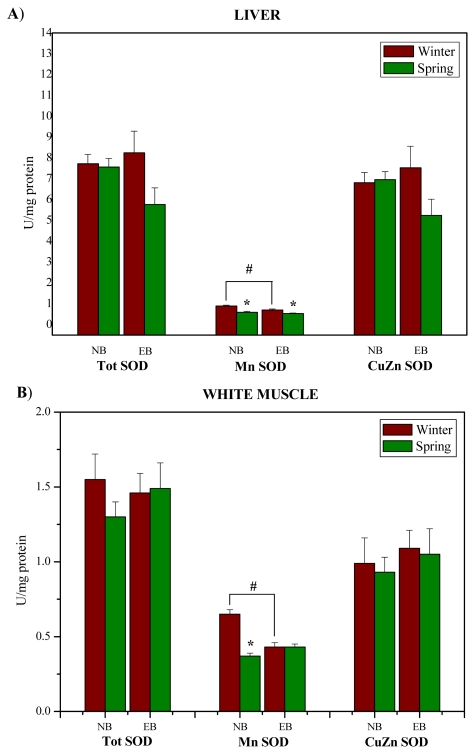
The activity (U/mg protein) of Tot SOD, CuZn SOD and Mn SOD in the liver (A) and white muscle (B) of red mullet (*M. barbatus*) from the Near Bar (NB) and Estuary of the River Bojana (EB) in winter and spring. The data are expressed as mean ± S.E. The non-parametric Mann-Whitney U-test was used to seek significant differences between means. * p < 0.05 represents a minimal significant level for effects of season; ^#^ p < 0.05 represents a minimal significant level for effects of site.

The obtained results of the activity of antioxidant defense enzymes and biotransformation phase II enzyme GST support the hypothesis of seasonal patterns of antioxidant defense enzymes in the liver and white muscle of red mullet. Our results show that Mn SOD activity was significantly lower in spring in comparison to winter (p < 0.05) in the liver (Figure 2A) at both examined localities, and in white muscle (Figure 2B) in NB locality. In addition, Mn SOD activity was significantly lower in spring compared to winter at EB than at NB (p < 0.05) in both the liver and the white muscle (Figure 2A and 2B).

**Figure 3:**
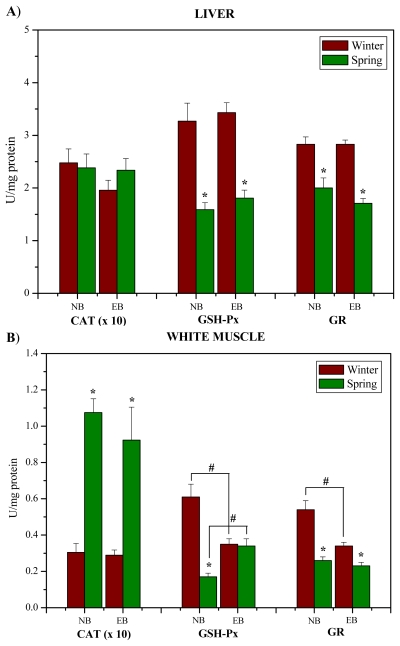
The activity (U/mg protein) of CAT, GSH-Px and GR in the liver (A) and white muscle (B) of red mullet (*M. barbatus*) Near Bar (NB) and Estuary of the River Bojana (EB) in winter and spring. * p < 0.05 represents a minimal significant level for effects of season; ^#^ p < 0.05 represents a minimal significant level for effects of site.

The activity of CAT in white muscle (Figure 3B) was significantly higher in spring in comparison to winter at both investigated localities (p < 0.05). The activities of GSH-Px and GR in the liver (Figure 3A) and white muscle (Figure 3B) were markedly lower in spring than in winter at both investigated localities (p < 0.05), except muscle GSH-Px activity at EB (Figure 3B). In addition, muscle GSH-Px and GR activities were markedly lower at EB than at NB in winter (p < 0.05), and GSH-Px activity was higher at EB than at NB in spring (p < 0.05), (Figure 3B).

**Figure 4:**
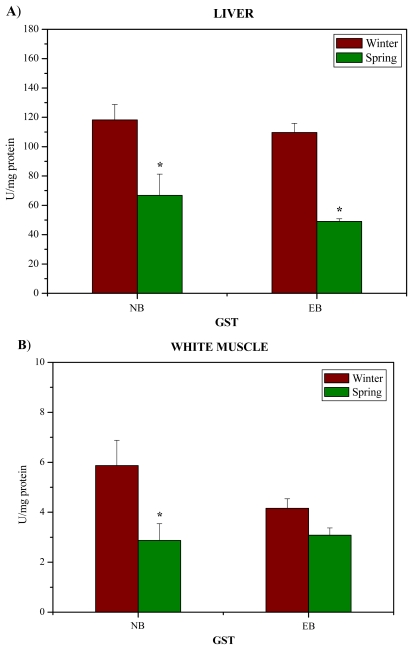
The activity (U/mg protein) of GST in the liver (A) and white muscle (B) of red mullet (*M. barbatus*) from the Near Bar (NB) and Estuary of the River Bojana (EB) in winter and spring. * p < 0.05 represents a minimal significant level for effects of season.

The activity of biotransformation phase II enzyme GST was considerably lower in spring compared to winter at NB and EB localities (p < 0.05) in the liver (Figure 4A), as well as at NB locality in the white muscle (Figure 4B).

In an aquatic environment, despite the presence of constitutive or enhanced antioxidant defense systems, increased levels of oxidative damage will occur in the organisms exposed to contaminants that stimulate the production of ROS [21]. The responses of fish to a variety of metal and organic pollutants are transient and are dependent on the species, enzymes and single or mixed contaminants. The responses of biomarkers can be masked by the nutritional status of the animals and it has been proposed that the animals inhabiting chronically polluted environments can develop some adaptation or compensatory mechanisms [22].

Studies on antioxidant defense enzyme activities related to seasonal changes in poikilothermic organisms revealed its strong relationship with metabolic demands [5,9]. It means that lower metabolic rate is accompanied with lower antioxidative defense, but the role of individual components in achievement of homeostasis seems to be different and integrated in antioxidative defense system. Fish are exposed to daily and/or seasonal changes in both water temperatures and oxygen availability and variations in the activity of antioxidant defense enzymes have been demonstrated in several studies and proposed as biomarkers of pollutant-mediated oxidative stress [18,22].

In marine water, dominant differences between winter and spring are temperature (higher in spring) and concentration of dissolved oxygen (greater in winter). The major antioxidative defense enzymes in marine fish are SOD, CAT and GSH-Px [4]. Antioxidative status in species of marine fish seems to be related to tissue oxygen consumption or to organism activity level [7]. Many studies have shown the differences in both behavior and biochemical parameters with respect to environmental temperature. In autumn and winter, individuals of medaka (*Oryzias latipes*) became less active and showed relatively higher activity at night [23]. In other marine organisms, such as mussels, *Mytilus galloprovincialis,* a marked reduction in the antioxidative defense system occurred during winter [24]. This may be associated with changes in environmental temperature, as well as in gonad maturation and food availability. Many other enzymes have reduced activities at lower environmental temperature: xanthine dehydrogenase activity in mussels from the Atlantic Ocean [25], GST activity in viviparous blenny, *Zoarces viviparus* in the Baltic Sea [26]. However, some enzymes increase their activities in winter, e.g., etoxycoumarin and etoxyresorufin O-dealkylases in red mullet, *Mullus barbatus* [27]. Sheehan and Power [13] conclude that the use of bioindicators, such as enzyme activities, in biomonitoring studies is often complicated, because levels of chemical pollutants in the environment often display wide seasonal variations in response to climate and other factors. Where such molecules show seasonal variation, this should be incorporated into the interpretation of biomonitoring studies by the use of appropriate controls.

Our previous investigations at the same localities [28] showed no significant differences in concentrations of polychlorinated biphenyls (PCBs) in both seasons. It is difficult to predict the direct influence of toxic compounds on antioxidant defense enzyme activities, because the situation is complicated with seasonal influences. It is well known that in aquatic ecosystems, temperature and dissolved oxygen are environmental variables that are likely to influence oxidative processes, even more than xenobiotics.

The overall trend obtained in our study, revealed decreased activities of the investigated enzymes in spring when compared to winter. Proteins constitute a target for oxidative damage with subsequent alteration of their functions. Studies by other authors reported that flounders, living in contaminated waters with xenobiotics, showed increased levels of oxidized proteins [29]. The major difference in our work was found for Mn SOD activity in the liver and white muscle of red mullet, suggesting that in mullets, the liver mitochondria could efficiently deal with the increase in superoxide anion radicals [30]. It has to be referred that the food uptake can have an effect on antioxidant defense enzyme activities and oxidative stress, as the fish do not eat during the depuration period, as Pascual *et al*. [31] showed in *Sparus aurata*. As the lipid storage is mobilized to cope with the metabolic needs, lipids become the target that is more exposed to oxidation. Indeed, an increase in SOD activity in fasting fish was reported by the same authors. At the same time, all investigated glutathione-dependent enzymes (GSH-Px, GR and GST) showed decreased activities in spring in respect to winter. At low temperatures, the increased polyunsaturation of mitochondrial membranes in fish should raise rates of mitochondrial respiration, which would in turn enhance the formation of ROS, increase proton leak and favor peroxidation of these membranes. The mitochondria show seasonal cycles of the maximum rates of protein-specific substrate oxidation at any given temperature. Increases in the maximal capacity of pyruvate oxidation were sufficient to compensate for seasonal changes in temperature, except during the winter months when rates at habitat temperature were approximately half the rates over other periods [32]. In addition, higher levels of organic hydroperoxides are formed by enhanced lipid mobilization, which leads to induction of higher GSH-Px activity. This induces enhanced utilization of GSH, forming their oxidized form (GSSG), and consequently, influences elevated activity of GR in order to maintain sufficient amount of reduced equivalents in the cells and thus normal redox homeostasis. Induction of GR activity has been reported in various field studies in fish exposed to organic pollutants, such as PAHs, PCBs and halogenated xenobiotics [8,33]. Glutathione-S-transferases are a family of dimeric multifunctional enzymes that are shown to have been involved in detoxification of xenobiotics, protection from oxidative damage and the intracellular transport of hormones, endogenous metabolites and exogenous chemicals in diverse organisms [34]. However, there is some information regarding sexual, seasonal and developmental differences in GST activity in fish [35]. Some findings show that biotransformation phase II enzyme GST is influenced by the levels of organic substrates and both enhancement and inhibition of these enzymatic activities were reported in field studies [19]. Our results show that GST activity was higher at both sites in winter. These data suggest that GST enzyme is more reactive to the organic pollutants in winter, thus being a sensitive and suitable marker of environmental status, especially in the liver of red mullet. In winter, possible effects of organic pollutants on GST activity are more pronounced, according to the synergism between cold-stress and toxic effects and chemical pollutants [36]. The changes in the activity of antioxidant defense enzymes observed in red mullets in different seasons confirm that the animals exposed to oxidative stress can reprogram the cell response in changed environmental conditions.

**Figure 5:**
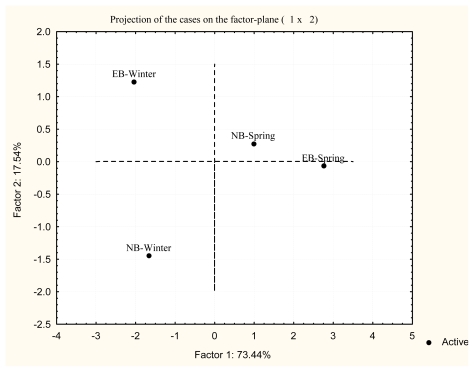
Principal Component Analysis (PCA) of antioxidant defense enzyme activities in the liver at each site and in season on the factor plane.

**Figure 6:**
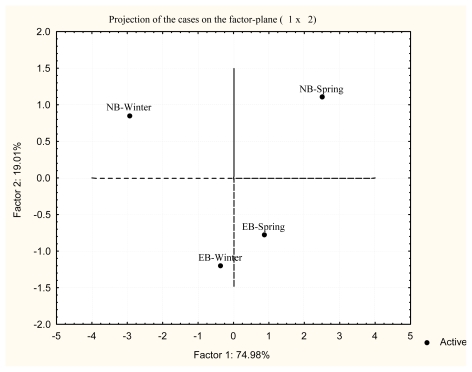
Principal Component Analysis (PCA) of antioxidant defense enzyme activities in the white muscle at each site and season on the factor plane.

Principal Component Analysis (PCA) was applied in order to statistically define the differences of antioxidative defense enzyme activities between the two investigated localities Near Bar (NB) and Estuary of the River Bojana (EB) in winter and spring. The results of PCA of the investigated antioxidative defense enzyme activities and biotransformation phase II enzyme GST are presented in Figure 5 for the liver and in Figure 6 for the white muscle of red mullet.

The treatment of overall data by PCA indicated a clear separation of various sampling periods for both tissues and a marked difference between seasons. A balanced action of antioxidative components is necessary for homeostasis of ROS and redox state. Changes in the activity of some antioxidant components should be accompanied by correlative changes in other. Examination of seasonal pattern of antioxidant defense enzyme activities and biotransformation phase II enzyme GST in the liver revealed clear differences in their activity (Factor 1: 73.44% and Factor 2: 17.54%) between winter and spring at both localities (Figure 5). Similar results were obtained for the white muscle (Factor 1: 74.98% and Factor 2: 19.01%) between winter and spring at both localities (Figure 6). The projection was made for all enzyme activities of each season based on the factor plane. Additionally, apart from seasonal differences, PCA in the white muscle clearly shows the differences between sampling localities as well.

Marine ecosystems represent the ultimate sink of both natural and anthropogenic inputs of contaminants. In order to prevent these events, a battery of biomarkers has been used as effective early warning tools in ERA and marine environment monitoring [37]. Several studies have shown that the physiological status of marine organisms changed when exposed to contaminants. One of the consequences was a lowered ability of organisms to tolerate the natural fluctuations of environmental factors. The annual variation of a particular biomarker response should be known and well understood prior its use in biomonitoring studies in order to separate successfully the contamination effects from the effects caused by normal physiological variations, and thus to interpret the results correctly.

## 3. Materials and Methods

### 3.1. Site description and sample collection

Red mullet (*Mullus barbatus* L.) were caught by trawling in winter (February) and late spring (May) at two localities: Near Bar (NB) and Estuary of the River Bojana (EB) in the Southern Adriatic Sea.

The two localities were chosen in order to compare the activity of antioxidant defense enzyme activities between periods of low metabolic activity in winter and basal metabolic activity in spring. These areas have similar climatic conditions, with the lowest mean water temperature in February and highest in August. The bottoms of the biotopes are covered with thick stratum of fine terrigenous mud containing particles of detritus. Both localities receive extensive urban and industrial wastewater discharges [9,38]. From each location, Near Bar and Estuary of the River Bojana, 10 (5 in the winter and 5 in the spring) specimens of red mullet were collected.

### 3.2. Measurements of environmental parameters

Measurements of environmental parameters (temperature, salinity, oxygen concentration and oxygen saturation of seawater) were performed with a WTW (Wissenschaftlich-technische Werkstatten, Dr Karl Slevogt straße, Weilheim, Germany) multilab system. They were made at the time of fish sampling at the depths presented in Table 1.

### 3.3. Tissue preparation

Specimens of red mullet (*Mullus barbatus* L.) were collected and immediately transferred to seawater tanks. Individuals of the same size class weighing 50–70 g were selected to ensure uniformity of samples. Fish were killed by severing the spinal cord and dissected within 3 minutes on ice. The liver and white muscle were rapidly dissected, washed in ice-cold 0.65% NaCl and frozen in liquid nitrogen (−196 °C) before storage at −80 °C. The tissues were ground and homogenized in 5 volumes of 25 mmol/L sucrose containing 10 mmol/L Tris-HCl, pH 7.5 at 1500 rpm [39] using Janke & Kunkel (Staufen, Germany) IKA-Werk Ultra-Turrax homogenizer at 4 °C [40]. The homogenates were sonicated for 30 s at 10kHz on ice to release enzymes [41] and sonicates were then centrifuged at 4 °C at 100,000 g for 90 min. The resulting supernatants were used for further biochemical analyses.

### 3.4. Protein concentration measurements

Protein concentration in the supernatant was determined according to the method of Lowry *et al*. [42] and expressed in mg/g wet mass.

### 3.5. Determination of antioxidant defense enzyme activities

The activity of antioxidant defense enzymes was measured simultaneously in triplicate for each sample using a Shimadzu UV-160 spectrophotometer and a temperature-controlled cuvette holder. The activity of total SOD was determined by the epinephrine method [43] and expressed as U/mg of protein. For the determination of Mn SOD activity, the assay was performed after the pre-incubation with 8 mmol/L KCN. CuZn SOD activity was calculated as a difference between total SOD and Mn SOD activities. CAT activity was assayed by the rate of hydrogen peroxide (H_2_O_2_) decomposition and expressed as μmol H_2_O_2_/min/mg protein [44]. The activity of GSH-Px was determined following the oxidation of nicotineamide adenine dinucleotide phosphate (NADPH) as a substrate with t-butyl hydroperoxide [45] and expressed in nmol NADPH/min/mg protein. GR activity was measured as described by Glatzle *et al*. [46] and expressed as nmol NADPH/min/mg protein. The activity of biotransformation phase II enzyme GST towards 1-chloro-2,4-dinitrobenzene (CDNB) was determined by the method of Habig *et al*. [47] and expressed as nmol GSH/min/mg protein. All chemicals were the products of Sigma (St. Louis, MO, USA).

### 3.6. Statistical analysis

All data values are given as the mean ± S.E (standard error). Statistical analysis was performed using the non-parametric Mann-Whitney U-test to seek significant differences between the means. A minimum significance level of p < 0.05 was accepted. Principal Component Analysis (PCA) was employed to detect variables that significantly contributed to differences in the activity of the investigated enzymes between the examined seasons. Analytical protocols described by Darlington *et al*. [48] and Dinneen and Blakesley [49] were followed.

## 4. Conclusions

Our study is the first comprehensive report of antioxidant defense enzyme activities in the red mullet, *Mullus barbatus*, collected from the investigated localities from the Montenegrine coastline in the Adriatic Sea. The results obtained in this study indicate a significant influence of seasonal factors on the activities of SOD, CAT, GSH-Px and biotransformation phase II enzyme GST in the liver and white muscle of red mullet (*Mullus barbatus*). The treatment of overall data by PCA indicated a clear separation of various sampling periods for both investigated tissues and a marked difference between seasons. Therefore, it can be concluded that seasonal factors should be incorporated into interpretation of mullet-based biomonitoring studies. Oxidative stress parameters and biotransformation phase II enzyme GST serve as good biomarkers of oxidative perturbations in this bioindicator species. The activity of antioxidant defense enzymes investigated in this work should be taken into account in further biomonitoring studies in fish species and adequately considered when biomarker responses are interpreted to detect anthropogenic disturbance. Our results are in accordance with similar monitoring studies and represent a further support in the assessing the health of coastal areas, and also the suitability of *Mullus barbatus* as a sentinel species in the future field studies in the Adriatic Sea.

## Figures and Tables

**Table 1:** Physic-chemical parameters of the sea water (temperature, salinity, O_2_ concentration and O_2_ saturation) at the examined locations (Near Bar - NB and Estuary of the River Bojana - EB) in winter and spring.

*Location*	*Season*	Temperature(°C)	Salinity (‰)	O_2_ concentration (mg/L)	O_2_ saturation
**NB**	**Winter**	11.60	32.85	8.30	91.0
	**Spring**	19.37	37.97	7.13	101.3

**EB**	**Winter**	11.67	37.67	8.37	94.3
	**Spring**	18.57	37.20	7.63	103.0

**Table 2:** Total protein concentration (mg/g wet mass) in the liver and white muscle of red mullet (*Mullus barbatus* L.) from the Near Bar (NB) and Estuary of the River Bojana (EB) in winter and spring. The data are expressed as mean ± S.E. The non-parametric Mann-Whitney U-test was used to seek significant differences between means.

*Location*	*Season*	LIVER	WHITE MUSCLE
**NB**	**Winter (n=5)**	315.12 ± 17.58	122.02 ± 9.17
	**Spring (n=5)**	306.70 ± 11.63	162.42 ± 8.19*

**EB**	**Winter (n=5)**	394.90 ± 13.17	167.76 ± 9.46
	**Spring (n=5)**	339.34 ± 3.31*	151.20 ± 8.67

* p < 0.05 represents a minimal significant level for effects of season.
